# Turkish Adaptation and Psychometric Evaluation of the Barriers to Incontinence Care-Seeking Questionnaire

**DOI:** 10.1007/s00192-025-06199-7

**Published:** 2025-07-07

**Authors:** Seda Yakit Yeşilyurt, Hanife Büşra Hekimoğlu, Merve Başol Göksülük, Patricia Brihuega González, Hatice Çankaya, Nuriye Özengin

**Affiliations:** 1 Faculty of Health Sciences, Department of Physiotherapy and Rehabilitation, Izmir University of Economics, Izmir, Turkey; 2 Institute of Postgraduate Education, Bolu Abant Izzet Baysal University, Bolu, Turkey; 3 Faculty of Medicine Department of Biostatistics, Sakarya University, Sakarya, Turkey; 4 NHS Isle of Wight Trust, Newport, UK; 5 Bolu Provincial Health Directorate, Refika Baysal Community Health/Healthy Life Center, Bolu, Turkey; 6 Faculty of Health Sciences, Department of Physiotherapy and Rehabilitation, Bolu Abant Izzet Baysal University, Bolu, Turkey

**Keywords:** Obstacle, Pelvic floor dysfunction, Questionnaire, Translate

## Abstract

**Introduction and Hypothesis:**

This study was aimed at adapting, validating, and assessing the reliability of the Turkish version of the "Barriers to Incontinence Care-Seeking Questionnaire" (BICS-Q).

**Methods:**

One hundred and forty-eight Turkish women with mild to moderate urinary incontinence (UI) was assessed. The adaptation process was conducted in alignment with the COSMIN guidelines: forward–backward translation, expert review, cultural adaptation, and a pilot study. For construct validity, items from the study by El-Azab and Shaaban, which measure barriers to seeking care for incontinence, were adapted to this self-reported questionnaire (BICS-Q), and their associations with the total score as well as the subscale scores of the questionnaire were evaluated. Cronbach's alpha was used for internal consistency, and the intraclass correlation (ICC) coefficient was estimated for test–retest reliability.

**Results:**

The psychometric analyses indicated that the Turkish BICS-Q has high internal consistency (Cronbach's alpha = 0.846) and test–retest reliability (ICC = 0.854). Item analysis revealed that each item was significantly correlated with the total score, thereby confirming construct validity. It was also found that embarrassment, financial concerns, and low expectations from medical consultation were ranked as the most important barriers to treatment.

**Conclusions:**

This study demonstrated the applicability of the Turkish BICS-Q for both research and clinical purposes, emphasizing its role in identifying health care inequalities and guiding policy improvements for women with UI. The present validation study provides evidence that the Turkish BICS-Q is an appropriate tool for assessing barriers to incontinence care seeking that can be used to further research and interventions toward overcoming the barriers in Turkey.

## Introduction

Urinary Incontinence (UI) is defined as"involuntary loss of urine"in the 2010 standardization report by the International Continence Society (ICS) [1]. In women, the prevalence of UI is 25–45%, and this rate increases with age [2]. According to the World Federation of Incontinent Patients, 50% of women report experiencing incontinence at some point in their lives [3]. Although UI is not a life-threatening or dangerous condition, it can cause embarrassment, social withdrawal, and a reduction in quality of life. In elderly individuals, it is a significant cause of disability and dependence [4]. UI affects various aspects of life, including health, psychosocial well-being, work, and leisure in women [5]. Additionally, the personal and financial costs of UI are substantial. As reported by the International Consultation on Incontinence, the management of urinary incontinence follows a structured, stepwise approach—ranging from conservative lifestyle modifications to invasive surgical interventions—beginning with initial treatment strategies and advancing toward specialized care when necessary [6]. The treatment goals are to reduce incontinence episodes, the amount of urine leaked and nocturia, reduce the costs associated with pad usage, and improve quality of life by enabling the individual to engage in social activities again. Despite the significant impact that UI has on women's quality of life, the number of women seeking treatment is quite low [7]. Barriers to seeking treatment for UI in women may stem from both internal and external factors. Many studies have explored internal barriers, such as shame and the normalization of the condition [8, 9]. External factors, or barriers, are different from internal attitudes such as shame, fear, or anxiety, and refer to specific, objective external conditions that prevent or hinder treatment-seeking behavior [10]. Literature indicates that barriers to seeking treatment for UI include ethnic differences, high costs, low socioeconomic status, and lack of health care services [11, 12]. In a study by Fathy et al. on elderly adults with UI, the most significant barrier to treatment seeking was the high cost of treatment and the lack of insurance coverage for these treatments [13]. Insufficient health care services or the indifference of health care professionals are also major barriers to treatment for women with UI [14]. Removing the barriers to treatment seeking in women is crucial for addressing health care inequalities in this population. However, there is no valid and reliable questionnaire in Turkish to assess barriers to treatment-seeking in women with UI. The Barriers to Incontinence Care-Seeking Questionnaire (BICS-Q), developed by Heit et al., is a valid and reliable tool for evaluating external barriers to treatment seeking [10]. This questionnaire has only been cross-culturally adapted to Spanish [15]. Considering the need in this area, the purpose of the planned study is to test the validity and reliability of the Turkish version of the BICS-Q.

## Materials and Methods

### Participants

The sample size for the study was determined as 148 participants, based on the guideline of having at least 10 participants per item for the 14-item Barriers to Incontinence Care-Seeking scale [16]. Women were invited to participate via personal networks of the researchers and announcements on social media (Instagram, Facebook, X, etc.). After providing verbal and written information about the study, signed informed consent forms were obtained from the participants. The study was approved by the Ethics Committee of Bolu Abant Izzet Baysal University (Decision No: 2022/60, Date: 8 March 2022). Women who volunteered were over 18 years old, literate in Turkish, and had UI symptoms. Exclusion criteria were the presence of neurological diseases, inability to complete the surveys owing to language or cognitive limitations, pelvic organ prolapse in stages greater than 2, recurrent urinary tract infections, predominant voiding dysfunction, and failure to participate in the second evaluation. The study methodology and report followed Consensus-based Standards for the Selection of Health Measurement Instruments guidelines.

### Translation

Before translating the BICS-Q into Turkish and performing validity and reliability studies of the Turkish version, permission was obtained from the authors via e-mail. The adaptation of the scale into Turkish was carried out in a five-stage protocol [17, 18]. The first stage of the translation process involved translating the English-language scale into Turkish. The translation was done by researchers who were fluent in both languages, knowledgeable about the field, and native Turkish speakers. The translation was made in accordance with the structure and format of the original English scale. In stage 2 (Synthesis), a jury proficient in both languages (English and Turkish) reviewed the translation to identify any inadequate terms or expressions, and the final version of the translated scale was completed. In stage 3, the scale, initially translated into Turkish and finalized by the jury, was re-translated into English by a translator with no knowledge of the subject. In stage 4, an expert committee of health care professionals (physiotherapist and obstetrician and gynecologist) conducted a discussion to ensure cultural equivalence and minimize differences between the Turkish and English versions. Issues such as synonymous words, grammatical problems, colloquial expressions, idiomatic difficulties, and the use of terms appropriate for Turkish cultural lifestyles were discussed. The final version of the scale was prepared before the pilot study was conducted. In the final stage (stage 5), after ensuring equivalence between the re-translated English version and the original scale, the Turkish version of the scale was applied to a population of 30 individuals with UI. The population consisted of individuals aged 18 and above, from different socioeconomic backgrounds, literate, and with no comprehension issues. These individuals were asked if they had difficulty understanding any expressions or complex terms in the scale, and any unclear words were explained and repeated. Suggestions regarding words were noted and considered, and expressions were changed to those more commonly used in Turkish culture. After completing all cultural adaptation procedures and making corrections, the final version of the scale was prepared.

### Measurement

Physical characteristics of the participants (age, body weight, height), educational status, menstrual status, obstetric history, chronic diseases, pelvic trauma, and surgical history, smoking habits, income level, socioeconomic status, social security, and obesity information were recorded. The presence and type of UI were determined using the 3-Incontinence Questionnaire (3IQ), which consists of three questions. The first question determines the presence of incontinence, whereas the second and third questions assess stress urinary incontinence (SUI), urge urinary incontinence (UUI), mixed urinary incontinence (MUI), or other types of incontinence [19].

To assess incontinence symptoms and quality of life, the International Consultation on Incontinence Questionnaire-Short Form (ICIQ-SF) was used. The ICIQ-SF was developed by Avery et al. [32] to assess incontinence symptoms and the impact of incontinence on quality of life. Internal consistency measurements have shown that the ICIQ-SF provides highly reliable, consistent, stable, and reproducible data, with high levels of sensitivity, reliability, and validity. The four-dimensional scale assesses the frequency of incontinence (dimension 1), the amount of urine leaked (dimension 2), the impact of incontinence on daily life (dimension 3), and when incontinence occurs (dimension 4). The first three dimensions are scored, and the responses to dimension 4 are used to identify the type of incontinence. The ICIQ-SF score is obtained by summing the scores of the first three dimensions. The score can range from 0 to 21, with higher scores indicating a greater impact of incontinence on quality of life [20].

Incontinence severity was assessed using the Incontinence Severity Index (ISI), which consists of two items. The first item assesses the frequency of incontinence, and the second item assesses the amount of urine leaked. The total score is calculated by multiplying the scores of the two items, with a score range from 1 to 12. A higher score indicates more severe UI. Studies have shown that the ISI is a valid measure for assessing incontinence severity [21].

To enhance the cultural relevance of the questionnaire and strengthen construct validity, the seven additional items proposed by El-Azab and Shaaban [22] were included alongside the original BICS-Q items. These items have been previously used to assess barriers to incontinence care seeking in Middle-Eastern populations and were considered culturally compatible with the Turkish context. Women were asked to respond to these seven statements using the following options:"not at all (0),""to some extent (1),"and"to a great extent (2)."The seven statements are:

1. Embarrassed to discuss my problem with any health care provider (I1)
2. There is no need for medical help because incontinence is a normal consequence of aging (I2)
3. I prefer to discuss the problem with relatives or friends (I3)
4. Urinary incontinence may resolve spontaneously (I4)
5. I have low expectations from medical consultation (I5)
6. The health care provider will not be interested in my problem (I6)
7. I think the financial cost of solving my problem will be too high (I7)

Participants were asked,"Have you sought treatment for incontinence?"For those who had not sought treatment, they were asked to explain in their own words,"What has prevented you from seeking treatment for your incontinence problem?"The responses provided by women who did not seek treatment to the open-ended question were categorized into three main domains: internal barriers, emotional/cultural barriers, and medical barriers. Finally, the BICS-Q was administered to assess barriers to treatment seeking. The BICS-Q consists of a 14-item scale, including items related to Inconvenience, Relationships, Site-related, Cost, and Fear. The total BICS-Q score is calculated by summing the subscale scores. Higher barrier scores are associated with a decreased likelihood of seeking care for incontinence. The BICS-Q was repeated 1 week later for test–retest reliability analysis.

### Statistical Analyses

For descriptive statistics, the mean ± standard deviation or median and minimum–maximum values were provided for numerical variables, and frequency and percentage values were given for categorical variables. The Shapiro–Wilks test and graphical methods (box plot, histogram, etc.) were used to assess normality.

For construct validity, the items suggested by El-Azab and Shaaban [22] for assessing barriers to incontinence care seeking were applied to the participants, and the relationships between each item and the total score as well as subscale scores were examined.

Confirmatory factor analysis (CFA) was conducted to assess the consistency of the adapted scale with the original version. Among the goodness-of-fit indices, root mean square error of approximation (RMSEA), minimum discrepancy function divided by degrees of freedom (CMIN/DF), goodness-of-fit index (GFI), and comparative fit index (CFI) were examined. Lower RMSEA values indicate a better fit, with values ≤ 0.08 considered good. CMIN/DF values ≤ 3 are generally interpreted as acceptable, whereas GFI and CFI values ≥ 0.90 indicate a good model fit. Differences in demographic and clinical characteristics of the BICS-Q score are another method used to assess construct validity. To examine whether there are differences between groups, either one-way analysis of variance (ANOVA) or the Kruskal–Wallis test was employed. Post-hoc tests were then used to identify the specific group or groups responsible for any significant differences.

To assess the reliability of the scale, Cronbach's alpha coefficient was calculated for both subscales and the total scale to examine whether each item in the scale measures the same construct. A Cronbach's alpha coefficient of 1.00 to 0.80 was considered to indicate a high level of reliability, 0.79 to 0.60 indicated acceptable reliability, 0.59 to 0.40 indicated a low level of reliability, and a coefficient below 0.39 was considered unreliable. To evaluate internal consistency, the scale was applied twice to the participants, and consistency was assessed using the intraclass correlation (ICC) coefficient. An ICC coefficient greater than 0.75 was interpreted as excellent, between 0.74 and 0.40 as moderate, and below 0.39 as poor reliability. With item analysis, the contribution of each item to the scale was evaluated using various measurement values. For this purpose, item-total correlation (corrected item-total correlation), multiple R-squared (squared multiple correlation), and Cronbach's alpha if the item was deleted (Cronbach's alpha if item deleted) were examined. All analyses were performed using IBM SPSS v.26. A significance level of *p* < 0.05 was considered.

## Results

In this study, as the questionnaire consisted of 14 items, and considering a potential dropout rate of 10%, a total of 155 participants were included and 7 were excluded from the study. Three women were removed from the study because they did not answer all the questions, and 4 were excluded owing to incorrect data entries. The physical and sociodemographic characteristics of the 148 women included in the study are shown in Table 1.

**Table 1:** Physical and sociodemographic characteristics of the women (*n* = 148)

Variable	Data
Age (years), mean ± SD	52.06 ± 11.08
Height (cm), mean ± SD	160.62 ± 6.48
Body weight (kg), mean ± SD	73.21 ± 11.1
BMI (kg/m^2^), mean ± SD	28.57 ± 4.67
Smoking status, number (%)	
Yes	31 (21.2)
No	100 (68.5)
Quit	15 (10.3)
Education level, number (%)	
Illiterate	2 (1.4)
Primary school	44 (29.7)
Secondary school	16 (10.8)
High school	30 (20.3)
University	50 (33.8)
Master's degree	6 (4.1)
Employment status (employed), number (%)	55 (37.2)
Income level, number (%)	
None	31 (31.0)
Low	20 (20.0)
Middle	45 (45.0)
High	4 (4.0)
Social security (available)	136 (92.5)
Place of residence, number (%)	
Urban center	31 (31.0)
District center	27 (18.2)
Rural (village, pasture, etc.)	4 (2.7)
Menstrual status, number (%)	
Spontaneous menopause	67 (45.6)
Surgical menopause	13 (8.8)
Regular menstrual cycle	49 (33.3)
Irregular menstrual cycle	18 (12.2)
Total number of pregnancies, median (minimum – maximum)	3 (0–8)
Number of live-born children, median (minimum – maximum)	2 (0–6)
Number of miscarriages, median (minimum – maximum)	0 (0–4)
Number of abortions, median (minimum – maximum)	0 (0–3)
ISI, median (minimum – maximum)	2.5 (1–12)
ICIQ-SF, median (minimum – maximum)	7.0 (3–21)

*ISI* Incontinence Severity Index, *ICIQ-SF* International Consultation on Incontinence Questionnaire-Short Form, *BMI* body mass index

When women were asked whether they had sought treatment for incontinence, 18.2% (*n* = 27) reported that they had sought care. The responses of women who did not seek treatment were classified into three domains: internal barriers (e.g., neglect, postponement, being too busy, and not perceiving a need for treatment), emotional/cultural barriers (e.g., fear, embarrassment, unwillingness to undergo medical examination, and concerns related to the pandemic), and medical barriers (e.g., reluctance to use medication and concerns about contracting an infection during examination). Among those who had not sought treatment (*n* = 121), more than half (59.5%) cited emotional/cultural barriers, 38% reported internal barriers, and 1.7% indicated medical barriers as the reasons for not pursuing treatment.

Regarding the responses to the items proposed by El-Azab and Shaaban [22], participants' answers reflected both encouraging and concerning trends. On the positive side, 73.6% reported that they never felt embarrassed to share their issue with health care professionals, and 53.4% disagreed with the belief that incontinence is a normal part of aging that does not require treatment. Furthermore, 58.8% believed that the issue would not resolve spontaneously, 56.8% did not have low expectations from medical consultation, and 73% did not consider the financial cost of treatment to be a significant barrier. Conversely, some findings were less encouraging. A notable 70.2% preferred to discuss their incontinence problem with relatives or friends rather than health care professionals, and 79.1% believed that health care providers would not be interested in addressing their condition (Table 2).

**Table 2:** Barriers related to incontinence in women [22]

Statement	Not at all, number (%)	To some extent, number (%)	To a great extent, number (%)
1. Embarrassed to discuss my problem with any health care provider	109 (73.6)	30 (20.3)	9 (6.1)
2. There is no need for medical help because incontinence is a normal consequence of aging	79 (53.4)	45 (30.4)	24 (16.2)
3. I prefer to discuss the problem with relatives or friends	48 (32.4)	56 (37.8)	44 (29.7)
4. Urinary incontinence may resolve spontaneously	87 (58.8)	52 (35.1)	9 (6.1)
5. I have low expectations from medical consultation	84 (56.8)	46 (31.1)	18 (12.2)
6. The health care provider will not be interested in my problem	117 (79.1)	25 (16.9)	6 (4.1)
7. I think the financial cost of solving my problem will be too high	108 (73.0)	17 (11.5)	23 (15.5)

**Fig. 1:**
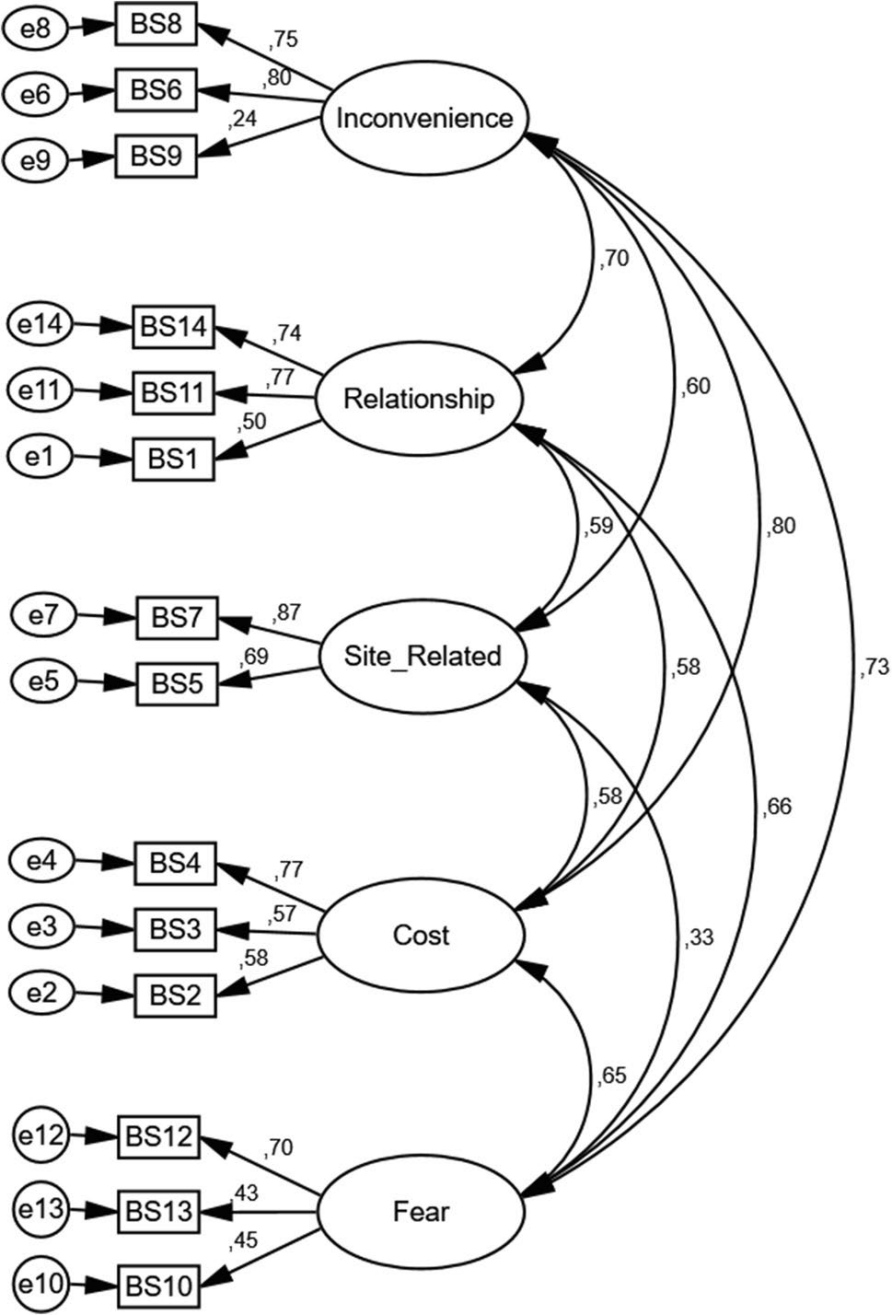
Confirmatory factor analysis results

The convergent validity of the adaptation to Turkish was assessed using a CFA method. After modification indices were examined, the final model was presented in Fig. 1. As shown in Fig. 1, all standardized factor loadings, except for item 9, fall within acceptable ranges (from 0.43 to 0.87). However, item 9 has a notably low factor loading, indicating that it does not significantly contribute to its designated subscale. Moreover, item 9 forms a separate factor on its own, suggesting that placing it within a different subscale would not impact the overall structure of the model. The decision to retain or remove item 9 was evaluated by experts in the field. Considering its clinical relevance, it was ultimately decided to keep the item in the scale. The CFA results indicated model fit indices close to acceptable thresholds: RMSEA = 0.10, CMIN/DF = 2.604, GFI = 0.86, and CFI = 0.82.

To evaluate the reliability and consistency of the proposed barriers scale, Cronbach's alpha coefficient and ICC coefficient results for both the total score and each subdimension are presented in Table 3. When examining the consistency levels of the items, Cronbach's alpha coefficients ranged between 0.542 and 0.747. The total score demonstrated a high level of reliability, with an alpha coefficient of 0.846. The ICC values for the test–retest results indicated moderate to high reliability.

**Table 3:** Examination of the reliability level of the barriers scale

Subscale	Cronbach's alpha coefficient	Intraclass correlation coefficient
Total score	0.846	0.854
Discomfort	0.616	0.829
Relationship	0.696	0.773
Location-related	0.747	0.753
Cost	0.648	0.828
Fear	0.542	0.808

An item analysis was conducted for all the questions included in the scale, and the results are presented in Table 4. When examining the correlation of each item with the total score, it was observed that all items, except for question 9, showed a correlation of 0.25 or higher. As removing question 9 would not result in a significant increase in Cronbach’s alpha coefficient (the coefficient would increase from 0.846 to 0.85 if the item were removed), it was decided that this question should remain in the scale, and the analysis proceeded without excluding it.

**Table 4:** Item analysis of the scale

Item	Corrected item-total correlation	Squared multiple correlation	Cronbach's alpha if item deleted
Question 1	0.422	0.348	0.840
Question 2	0.480	0.325	0.838
Question 3	0.460	0.349	0.839
Question 4	0.539	0.568	0.833
Question 5	0.448	0.447	0.839
Question 6	0.658	0.551	0.824
Question 7	0.554	0.532	0.833
Question 8	0.623	0.492	0.827
Question 9	0.219	0.188	0.850
Question 10	0.338	0.403	0.845
Question 11	0.606	0.472	0.828
Question 12	0.486	0.439	0.837
Question 13	0.372	0.321	0.843
Question 14	0.587	0.490	0.831

Analysis of the relationship between incontinence barrier items and scale scores was performed (Table 5). A significant difference was found between embarrassment when sharing the problem with health care professionals and the total score (*p* = 0.002), cost (*p* = 0.011), and fear (*p* < 0.001) subscales. A significant difference was observed between the perception of not needing treatment for UI and the total score (*p* = 0.003), discomfort (*p* = 0.011), cost (*p* = 0.022), and fear (*p* < 0.001) subscales. Preferring to share the UI problem with relatives or friends showed a significant relationship with the total score (*p* = 0.002), discomfort (*p* = 0.001), relationship (*p* = 0.031), and cost (*p* = 0.002) subscales. A significant difference was found between the belief that the UI problem would resolve on its own and the fear subscale (*p* = 0.041). The expectation level from medical treatment was significantly related to the total score (*p* = 0.001), relationship (*p* < 0.001), location-related (*p* = 0.019), and fear (*p* = 0.003) subscales.

**Table 5:** Relationship between incontinence barrier items and the barrier scale

		Total*	Discomfort	Relationship	Location related	Cost	Fear
Barrier items related to incontinence							
I1	Not at all	4^a^ (0–31)	2 (0—9)	0 (0—8)	0 (0—6)	0 (0—8)	0^a^ (0—9)
	To some extent	8^a, b^ (0–29)	2.5 (0—7)	2 (0—9)	0 (0—3)	0 (0—6)	2^b^ (0—6)
	To a great extent	11^b^ (7–24)	4 (2—6)	2 (0—7)	0 (0—5)	1 (0—6)	3^b^ (0–6)
	*p*	*0.002*	0.111	0.071	0.098	*0.011*	* < 0.001*
I2	Not at all	4^a^ (0–22)	1^a^ (0—9)	0 (0—7)	0 (0—6)	0^a^ (0—6)	0^a^ (0—6)
	To some extent	8^a, b^ (0–29)	3^b^ (0—9)	1 (0—9)	0 (0—6)	0^a, b^ (0—8)	2^b^ (0—6)
	To a great extent	10.5^b^ (0–31)	3^b^ (0—7)	2.5 (0—8)	0 (0—6)	0.5^a, b^ (0—6)	2^b^ (0–9)
	*p*	*0.003*	*0.011*	0.061	0.203	*0.022*	* < 0.001*
I3	Not at all	2^a^(0–31)	1^a^(0–9)	0^a^(0–8)	0 (0–6)	0^a^(0–6)	1 (0–7)
	To some extent	5.5^a^ (0–24)	2^a, b^(0–9)	1^a, b^(0–7)	0 (0–6)	0^a^(0–6)	1 (0–6)
	To a great extent	11.5^b^(0–29)	4^b^(0–7)	2^b^(0–9)	0 (0–6)	0.5^b^(0–8)	1.5 (0–9)
	*p*	*0.002*	*0.001*	*0.031*	0.150	*0.002*	0.102
I4	Not at all	5 (0–29)	2 (0–9)	1 (0–9)	0 (0–6)	0 (0–8)	0 (0–9)
	To some extent	7 (0–26)	2 (0–9)	1 (0–8)	0 (0–6)	0 (0–8)	1 (0–7)
	To a great extent	6 (0–31)	3 (0–7)	0 (0–8)	0 (0–6)	0 (0–3)	2 (0–7)
	*p*	0.827	0.964	0.921	0.318	0.758	*0.041*
I5	Not at all	3^a^(0–26)	1 (0–8)	0^a^(0–8)	0^a^(0–6)	0 (0–6)	0^a^(0–9)
	To some extent	8^b^(0–25)	2 (0–9)	2^b^(0–7)	0^a, b^(0–6)	0 (0–6)	2^b^(0–6)
	To a great extent	15^b^(0–31)	3 (0–9)	4^b^(0–9)	0.5^b^(0–6)	0.5 (0–8)	2^b^(0–7)
	*p*	*0.001*	0.101	* < 0.001*	*0.019*	0.080	*0.003*
I6	Not at all	4^a^(0–26)	2^a^(0–9)	0^a^(0–8)	0^a^(0–6)	0^a^(0–6)	0^a^(0–9)
	To some extent	11^b^(1–29)	3^b^(0–9)	2^b^(0–9)	1^b^(0–6)	0^a, b^(0–8)	2^b^(0–5)
	To a great extent	15.5^b^(9–31)	5^b^(3–7)	4.5^b^(1–8)	1.5^b^(0–6)	3^b^(0–6)	1^a^(0–7)
	*p*	* < 0.001*	*0.001*	*0.001*	*0.002*	*0.007*	* < 0.001*
I7	Not at all	3^a^(0–31)	1^a^(0–9)	0^a^(0–8)	0^a^(0–6)	0^a^(0–3)	0^a^(0–9)
	To some extent	13^b^(4–26)	4^b^(0–7)	2^b^(0–8)	1^b^(0–6)	2^b^(0–8)	3^b^(0–6)
	To a great extent	17^b^(6–29)	6^b^(0–9)	3^b^(0–9)	1^b^(0–6)	5^b^(0–8)	3^b^(0–7)
	*p*	* < 0.001*	* < 0.001*	* < 0.001*	* < 0.001*	* < 0.001*	* < 0.001*

Summarized using median (minimum, maximum)

Kruskal–Wallis test with Bonferroni correction was used

*I* item

* Letters (a, b) denote the results of post-hoc comparisons conducted following the Kruskal-Wallis test. Groups sharing identical letters (e.g., a a) are not significantly different from each other, whereas groups labeled with different letters (e.g., a b) exhibit statistically significant differences (*p *< 0.05)

Values with *p *< 0.05 are presented in italics

Variables that were considered to potentially affect the BICS-Q are presented in Table 6. There was a statistically significant difference between education level and the total (*p* = 0.033) and subscales score (discomfort, *p* = 0.040); location related, *p* = 0.008; and cost, *p* = 0.007). Similarly, employment status was significantly associated with the total (*p* = 0.006) and subscales score (relationship related, *p* = 0.022; location related, *p* < 0.001; and cost, *p* = 0.002). Participants who were unemployed had higher total and subscale scores. Income level was significantly associated with the total score (*p* = 0.002), discomfort (*p* = 0.005), location (*p* = 0.003), and cost (*p* = 0.001), with scores in both total and subscales decreasing as income level increased. Among the different places of residence, a significant difference was found only in the location-related subscale (*p* = 0.049), with participants living in city centers reporting lower scores. Regarding menstrual status, a significant difference was found in the total score (*p* = 0.033), discomfort (*p* = 0.029), and cost subscales. The highest scores were observed in the surgical menopause group, whereas the lowest scores were found in the regular and irregular menstruation groups. No significant associations were found between scale scores and smoking status, health insurance coverage, or hormone replacement therapy.

**Table 6:** Distribution of Barriers to Incontinence Care-Seeking Questionnaire (BICS-Q) total and subscale scores according to women's sociodemographic characteristics

Characteristic		Total score	Discomfort	Relationship	Location related	Cost	Fear
Education	Primary school	7.5^a, b^ (0–31)	2^a, b^ (0–9)	1^a, b^ (0–9)	0^a^ (0–6)	0^a, b^ (0–6)	0.5 (0–7)
	Secondary school	9.5^a^ (0–26)	4^a^ (0–7)	2^a^ (0–8)	0^a^ (0–6)	2^a^ (0–8)	1 (0–6)
	High school	7.5^a, b^ (0–25)	2.5^a, b^ (0–6)	1.5^a, b^ (0–7)	0^a, b^ (0–4)	0^a, b^ (0–8)	1 (0–9)
	Bachelor degree and postgraduate	3.5^b^ (0–22)	1.5^b^ (0–9)	0^b^ (0–8)	0^b^ (0–6)	0^b^ (0–5)	1 (0–6)
	*p*	*0.033*	*0.040*	0.389	*0.008*	*0.007*	0.967
Occupation	Unemployed	8 (0–31)	2 (0–9)	1 (0–9)	0 (0–6)	0 (0–8)	1 (0–9)
	Employed	4 (0–21)	2 (0–9)	0 (0–8)	0 (0–3)	0 (0–3)	1 (0–6)
	*p*	*0.006*	0.241	*0.022*	* < 0.001*	*0.002*	0.521
Income level	None	13^a^ (0–25)	3^a^ (0–9)	2^a, b^ (0–8)	0^a^ (0–6)	2^a^ (0–8)	2 (0–6)
	Low	7^a, b^ (0–29)	2.5^a, b^ (0–6)	1^a^ (0–9)	0^b^ (0–6)	0^a, b^ (0–6)	1 (0–9)
	Medium	4^b^ (0–31)	1^b^ (0–7)	0^a, b^ (0–8)	0^b^ (0–6)	0^b^ (0–3)	1 (0–7)
	High	3.5^b^(2–6)	2^a, b^(1–3)	1.5^b^(0–4)	0^a, b^(0–0)	0^a, b^(0–0)	0 (0–0)
	*p*	*0.002*	*0.005*	0.227	**0.003**	*0.001*	0.059
Residence	City	6 (0 – 26)	2 (0 – 9)	0 (0 – 8)	0 (0 – 6)	0 (0 – 8)	1 (0 – 9)
	District	8 (0 – 31)	2 (0 – 9)	2 (0 – 8)	0 (0 – 6)	0 (0 – 6)	1 (0 – 7)
	Rural Area	6 (1 – 29)	3.5 (1 – 6)	2.5 (0 – 9)	0.5 (0 – 3)	0 (0 – 6)	0.5 (0 – 5)
	*p*	0.269	0.758	0.063	**0.049**	0.792	0.769
Menstrual status	Spontaneous menopause	7^a, b^(0–31)	2^a, b^ (0–9)	1^a, b^ (0–9)	0^a^ (0–6)	0^a, b^(0–8)	1 (0–7)
	Surgical menopause	15^a^(0–26)	6^a^(0–6)	2^a^(0–7)	0^a^(0–6)	3^a^(0–6)	3 (0–9)
	Regular Menstruation	4^b^(0–21)	1^b^(0–9)	1^a, b^(0–8)	0^a, b^(0–4)	0^b^(0–6)	1 (0–5)
	Irregular Menstruation	3.5^b^(0–16)	2^a, b^(0–6)	0^b^(0–6)	0^b^(0–1)	0^b^(0–3)	0 (0–5)
	*p*	*0.033*	*0.029*	0.816	0.193	*0.003*	0.278

Descriptive statistics were presented as median (minimum–maximum)

Kruskal–Wallis test was used to compare the groups

Post-hoc test results were indicated by superscript letters (a, b). Identical letters (a, a) indicate no significant difference between groups, whereas different letters (a, b) indicate a statistically significant difference between groups

*BICS-Q* Barriers to Incontinence Care-Seeking Questionnaire

Values with *p* < 0.05 are presented in italics

## Discussion

The results of this study demonstrated that the Turkish version of the BICS-Q is valid and reliable for assessing barriers to seeking treatment for incontinence.

The BICS-Q was developed by Heit et al. is a valid tool designed to measure external barriers to care seeking. Melnyk’s Barriers Scale was developed to conceptualize barriers as consumers’ perceptions of costs or obstacles to accessing care. The BICS-Q consists of 13 items derived from Melnyk’s Barriers Scale and one additional item based on a review of medical literature and responses to open-ended questions from focus-group participants [10]. However, the BICS-Q had previously only been culturally adapted into Spanish [15], with no validation or reliability studies conducted in other languages, including Turkish. This study was aimed at addressing this gap and fulfilling the need for a Turkish adaptation.

For linguistic validation, the study used the forward–backward translation method, a standard approach widely applied in translation studies to ensure cultural and conceptual equivalence between the original and the target languages [23, 24]. This method enhances the comprehensibility of translated questionnaires for the target audience. Minor adjustments were made following comprehensibility interviews, such as adding “(treatment costs)” to item 2. Overall, the questions were found to be clear and straightforward in Turkish, and patients across different age and education groups could easily complete the survey.

The Spanish adaptation of the scale included only women over 65 years of age [15]. In contrast, the Turkish version included a broader, more heterogeneous sample of younger and middle-aged women, as in the original scale [10]. Additionally, in the Spanish version, the subsections related to cost and location were removed, and a new subsection addressing information deficiency was created. In the Turkish version, however, we believe that the item"Delays in the reimbursement of insurance (treatment expenses)"in the cost subsection may not be suitable for the current health system (such as the Social Insurance Institution, the Tradesmen and Craftsmen Social Security Institution, and Other Independent Workers Social Security Institution). However, since 2002, there has been a significant increase in private health care insurance in Turkey, and this growth trend will continue owing to the increasing share of the private sector in the health system [25]. Statistically, because removing the second item does not significantly change the Cronbach alpha coefficient, item 2 was used in the Turkish validity and reliability tests. The same applies to item 9. Although the correlation in the item analysis of item 9 is low (correlation item = 0.219), removing item 9 did not result in a significant increase in the Cronbach alpha coefficient (if the item were removed, the Cronbach alpha coefficient would increase from 0.846 to 0.85), and it was considered that this item should remain in the scale; thus, it was not removed.

El Azab and Shaaban reported that the current version of the scale is not suitable for assessing the barriers to seeking treatment for UI among Egyptian and likely Middle Eastern women. This is because the availability of free health care in state and university hospitals in Egypt was thought to invalidate the item related to health insurance (item 2) [22]. For this reason, the researchers created a seven-item questionnaire that evaluates barriers, which was also used in this study. However, as mentioned above, the private health insurance policies in modern Turkey and the Middle East differ [25, 26]. We believe that the approach to the items of the scale may stem from these differences.

The reliability method of the scale, test–retest reliability, refers to the ability of the scale to produce consistent results when applied to the same group again and to show stability over time [27]. For this, the scale was repeated for the same group of women with UI under the same conditions. The period between applications was determined to be 1 week, as it needed to be long enough to prevent significant recollections but short enough not to allow changes in the data being measured. The BICS-Q was applied to the same group twice, 1 week apart. During the test–retest period, significant correlation and agreement values were found. The results of the test–retest showed moderate to high reliability. These results demonstrate the strong internal consistency of the Turkish version of the BICS-Q. Additionally, 97.2% of the women participating in the study (144 participants) were reached for re-testing after 1 week, and a high rate of reliability testing was performed.

El Azab and Shaaban have identified the barriers to treatment seeking for UI in Middle Eastern women as a patriarchal society, feelings of shame, and the belief that this condition is a normal part of aging [7]. Therefore, they stated that the percentage of treatment seeking was very low, at about 4% [22]. However, cross-sectional studies conducted in our country have reported that the percentage of treatment seeking for UI problems ranges between 32 and 50% [28, 29]. This rate is reported to be 32% in Europe as well [30]. We believe that with increasing awareness of pelvic floor dysfunctions among women in Turkey in recent years, they may have overcome barriers stemming from internal factors, such as shame, viewing it as a normal condition, or fear, related to seeking treatment.

Studies have reported that women's treatment-seeking for UI is associated with the negative impact of UI on quality of life and the severity of incontinence, but there are also barriers such as the lack of awareness of the possibility of accessing health care and/or receiving treatment [31]. Barriers to seeking care for UI are external factors that prevent sufferers from seeking help, such as transportation or health insurance (external barriers). These differ from internal factors, i.e., attitudes that prevent sufferers from seeking care, such as embarrassment, fear, or anxiety (internal barriers). Identifying these barriers is crucial for evaluating inequalities in access to health care for the female population with UI [10].

The women with UI who participated in this study can be classified as having mild symptoms based on the ISI scale. Although it has been reported in the literature that treatment seeking for UI is associated with great symptom severity [31], the Turkish version of the scale can also be used to evaluate the barriers to treatment-seeking in women with mild UI.

There are several limitations to our study. First, the Turkish version of this scale is only suitable for the female population and does not examine treatment-seeking for UI in men. Another limitation is that the assessment of responsiveness of the Turkish version of the BICS-Q is not available. Although the BICS-Q assesses a broad range of structural and psychological barriers, it does not address deep-rooted sociocultural factors such as patriarchy, gender roles, and cultural stigma, which may be highly relevant in certain populations. This limitation should be considered in future adaptation or development of context-specific instruments.

Additionally, although the Turkish version of the BICS-Q was found to be culturally and conceptually appropriate, the results of the open-ended question revealed that a small number of participants reported barriers not explicitly addressed in the original questionnaire. These included concerns such as reluctance to use medication and fear of infection (particularly related to the COVID-19 pandemic). Although these responses were thematically similar to the existing BICS-Q subdomains, they reflect culturally specific nuances that may warrant further exploration. Therefore, although the BICS-Q is applicable to the Turkish context, future adaptations or supplementary tools may be necessary to fully capture context-specific sociocultural barriers to care-seeking behavior.

In conclusion, The Turkish version of the BICS-Q was found to be consistent, reliable, and valid for the Turkish population. It has been found that this scale can be used in both research and clinical studies. Additionally, identifying the barriers to treatment-seeking for UI in our country may offer a different perspective for health policies.

## Data Availability

This manuscript does not report data generation or analysis.
